# Inequality aversion and prosocial punishment: Evidence from a one-shot public goods game

**DOI:** 10.1371/journal.pone.0337425

**Published:** 2026-01-07

**Authors:** Per F. Andersson, Martina Testori, Sergio Lo Iacono

**Affiliations:** 1 Department of Political Science, Stockholm University, Stockholm, Sweden; 2 Department of Sociology, University of Essex, Colchester, United Kingdom; 3 Greenwich Business School, University of Greenwich, London, United Kingdom; Virginia Commonwealth University, UNITED STATES OF AMERICA

## Abstract

The willingness to engage in costly punishment of free riders (prosocial punishment) is crucial to foster group cooperation and understand public goods provision. While prosocial punishment is common across societies, its motivations remain unclear. Scholars have suggested that people resist inequitable outcomes and willingly bear costs to sanction free riders, seeking a fairer distribution of payoffs. This study tests a key implication of such fairness-driven arguments: if inequality aversion drives prosocial punishment, individuals should punish less when redistribution occurs, as equality concerns would be already satisfied. We conducted a pre-registered 2x2 between-subjects lab experiment (N=320), where participants completed a Social Value Orientation (SVO) task and played a one-shot Public Goods Game (PGG) with a Punishment Stage. We manipulated endowment inequality and the presence of redistributive taxation. Pre-registered analyses show that (1) inequality aversion does not predict prosocial punishment; (2) punishment levels do not significantly differ across treatments. However, exploratory results suggest that under high inequality, redistribution reduces the intensity of punishment towards richer individuals. This could indicate that inequality aversion triggers prosocial punishment only at acute inequality levels.

## Introduction

As documented by Piketty, 2014 [1] and Milanovic, 2016 [2], economic inequality has increased sharply in the last decades, reaching record levels in many countries. This is concerning since inequality has been linked to negative public health outcomes, reduced trust, increased crime, polarization, and political instability [3–8]. An even greater concern is that the reductions in extreme inequality have historically resulted from violence, as seen in events like the Russian Revolution of 1917 [9]. Although inequality threatens political stability, government redistribution can provide an effective and peaceful solution [10]. Understanding how inequality and redistribution may trigger or defuse sanctioning behaviors is thus key in order to address some of the fundamental political issues of our time.

The specific behavior we focus on in this paper is prosocial punishment, in which individuals pay a cost to sanction other individuals who harm or withhold benefits from others. While such behavior can create friction within communities, it also plays a crucial role in sustaining cooperation beyond close kin by enabling the enforcement of cooperative social norms. Many scholars view prosocial punishment as central to the evolution of large-scale human societies and relevant to understanding contemporary variations in the provision of public goods around the world [11–13]. When prosocial punishment is widespread, public goods are more readily provided, tax compliance is higher, and cooperation is more frequent [14–18]. In other words, even though excessive sanctioning can be harmful, its targeted use to enforce cooperative social norms appears to benefit society. The dual nature and broad implications of this behavior make it all the more important to understand its foundations at the individual level. Why do people engage in prosocial punishment behavior?

This question has been tackled by studies investigating human cooperation, often using game theory and laboratory experiments (for a review, see Kurzban and colleagues, 2015 [11]). A focal puzzle in this literature concerns the motives behind prosocial punishment. The challenge in accurately identifying the underlying motive behind this behavior stems from the fact that defection and inequality are often intertwined. When participants defect or free ride—in social dilemmas, they generate unequal payoffs. As a result, when analysts observe prosocial punishment, it can be difficult to determine whether it reflects a desire to equalize payoffs, a desire to harm the defector, or a combination of both. In other words, prosocial punishment in this setting can be explained both by (1) inequality aversion, that is, the willingness “to give up some material payoff to move in the direction of more equitable outcomes.” ([19], p. 819), and (2) negative reciprocity, that is, the sanctioning of defectors/free riders. As long as free riding leads to inequality and punishment reduces inequality, we cannot distinguish between negative reciprocity and inequality aversion.

In this paper, we present the results of an experiment that allows us to 1) study the impact of inequality and redistribution on prosocial punishment, and 2) better determine the degree to which this behavior is due to inequality aversion. We use a one-shot Public Goods Game (PGG) followed by a punishment stage and vary 1) the inequality of the initial endowment and 2) whether there is a redistribution stage before punishment, (Meltzer and Richard tax and transfer protocol is implemented [20]). We measure inequality aversion in two ways: first, directly, through the Social Value Orientation (SVO) survey [21], and second, indirectly, by observing behavioral patterns in the PGG. Our main findings are that, while neither inequality nor redistribution affects the total amount of punishment, they do affect the distribution of that punishment. When no redistribution is implemented in a high inequality setting, the higher the participants’ payoff, the greater the punishment they receive. However, this effect disappears when redistribution is introduced. Thus, when there is inequality, how punishment is administered changes if this inequality is partially addressed by redistributive policy. This means that part of the prosocial punishment behavior we observe could be explained by inequality aversion. Further, since we observe punishment even when removing a key driver for the inequality aversion motive, we speculate that the remaining sanctioning behavior may be due to negative reciprocity, the other main theoretical contender for this behavior. This does not exclude other potential channels, something we discuss in more detail in the Discussion section.

Our results are relevant for several different strands of research. Scholars in political science and economics have long studied how preferences for redistribution and taxation are formed. One of the key findings is that people generally support progressive taxation [7], and that inequality increases support for progressive taxation both in survey experiments [22] and in the lab [23]. Rueda and Stegmueller [24] find that inequality increases the support for redistribution even among the rich and argue that this is due to fear of crime. However, while much research is concerned with how inequality (and inequality aversion) affects preferences for taxation and redistribution, the reverse line of inquiry has received less attention. We therefore ask: how does redistributive taxation influence inequality aversion motives that may underlie sanctioning behaviors? This has clear policy implications: if inequality aversion drives engagement in punishment, it becomes important to understand how public policy can reduce reliance on such costly and potentially divisive behavior, while still achieving the goal of reducing inequality and increasing cooperation levels. We contribute to this literature by investigating how prosocial punishment is affected by both inequality and redistribution.

Our results are also of relevance for studies of human cooperation. Previous work has shown that people are ready to take on a personal cost to reduce the payoff of free riders [25], even in one-shot interactions [4]. Our results show that this behavior seems to be linked to inequality aversion under conditions of high inequality, but likely also involves other motives. By manipulating inequality in the initial endowments, our results also speak to studies showing how endowment inequality reduces contributions in public goods games (e.g., [26,27]).

In the next section, we discuss the theoretical background that motivates our hypotheses and experimental design. After that, we present the material and methods, as well as the statistical analysis employed.

### Theoretical background

Prosocial punishment occurs when participants willingly incur a cost to reduce the payoff of other players who harm or withhold benefits from others (i.e., defectors). This behavior is not compatible with simple self-centered utility maximization, since it lowers the final payoff of the punishers themselves. In a Public Goods Game (PGG), participants are asked to contribute a voluntary amount of their endowment to a common project, the total contribution is then multiplied and equally distributed among participants. Thus, the maximum payoff for an individual is achieved if the other participants contribute their entire endowment while she does not contribute at all. Previous research has found that, if given the opportunity (by adding a punishment stage after the PGG), participants routinely punish low contributions (reducing the payoff of defectors), even if this is costly for themselves [28,29]. This prosocial punishment has been shown to encourage cooperation in repeated games settings. Interestingly, this behavior is also common in situations in which there is no future cooperation to induce, such as in one-shot PGGs [4]. Thus, sustaining cooperation over time cannot be the only explanation.

There are two main motives suggested in the literature for why people engage in prosocial punishment in this context. The first is negative reciprocity (or retaliation), whereby people reciprocate a negative action with another negative response [30]. In this case, sanctioning defectors is a way to retaliate against free riders’ harmful behavior (even if sanctioning entails a cost). The second, inequality aversion, can be defined as the willingness “to give up some material payoff to move in the direction of more equitable outcomes” ([19], p. 819). As such, inequality aversion reflects a desire for a fair distribution of resources, the purpose of punishment is not to inflict damage on free riders, but to preserve equality. For a recent review of the literature that relates these to inequality and redistribution, see Dimick and colleagues’ work [31].

Numerous studies have found that inequality aversion motives correlate with prosocial punishment [32–35]. Fowler and colleagues [36] argue that innate human preferences for equality explains punishment better than negative emotions toward free riders and show that participants are more willing to punish high earners than low contributors. In this view, the goal of punishment is to produce a more equitable distribution of resources, not to foster cooperation or vent negative emotions towards defectors. This argument has received considerable attention in the literature, which generally finds that inequality aversion motives matter in influencing prosocial punishment [34,37]. However, inequality aversion is an individual preference and cannot be experimentally manipulated (for obvious ethical reasons). As a result, previous studies struggle to thoroughly rule out the effect of relevant confounders or establish whether the relationship is causal (rather than correlational). Furthermore, since defection leads to inequality in PGGs, it is difficult to disentangle punishment motives based on inequality aversion from punishment motives based on other rationales such as negative reciprocity [11,38,39]. Given such challenges, it is unsurprising that experimental research attempting to identify the role of inequality aversion in driving prosocial punishment reached mixed conclusions. Although a body of evidence suggests that negative reciprocity may be a stronger predictor of prosocial punishment than inequality aversion [38,40,41], a second branch of the literature argues that prosocial punishment more frequently stems from inequality aversion [33,42–44]. A related body of literature examines how inequality in endowments affects contributions in PGGs. Interestingly enough, Cherry and colleagues [26] find that contributions are lower in PGGs with endowment inequality and that this effect is not driven by whether the endowments are earned or not. The reduction in contributions in the inequality condition is due to the rich contributing less [27].

In this paper, we seek to advance the debate by testing the causal nexus between inequality aversion and prosocial punishment in the context of the PGG, addressing a key implication of fairness-driven arguments: if individuals punish free riders due to an aversion to inequality, then the presence of a redistributive institutional setting should reduce their propensity to sanction free riders, as their need to establish equality should have already been satisfied. Specifically, we suggest that third-party redistribution of resources following contributions to the public good will diminish the motivation for prosocial punishment among peers, thereby significantly reducing its frequency. Our pre-registered expectations can be formulated as follows. First, in line with previous research [33,34], we expect that (H1) participants with stronger inequality aversion (as measured by the SVO) will be more likely to exercise prosocial punishment across all conditions. Second, if redistribution moderates the association between individuals’ inequality aversion and prosocial punishment, then prosocial punishment will be less likely to occur when redistribution is applied. This should be robust and hold true regardless of the initial inequality, as heterogeneous contributions in the PGG will generate inequality in the distribution of resources across both conditions. Thus, we hypothesize that (H2a) when there is no inequality in the participants’ endowments, prosocial punishment in the No Redistribution treatment will be higher than in the Redistribution treatment for people with stronger inequality aversion, and that (H2b) when there is high inequality in the participants’ endowments, prosocial punishment in the No Redistribution treatment will be higher than in the Redistribution treatment for people with stronger inequality aversion. The pre-registration is available at https://osf.io/tq5gz/files/r3ykt.

To test these hypotheses, we conducted a pre-registered 2X2 between-subjects lab experiment (N = 320) where participants completed the SVO task and played a one-shot PGG followed by a punishment stage. We manipulated the presence of a redistribution stage (implemented after the PGG but before the punishment stage) and the level of endowment inequality (see Material and Methods for more details). Participants were informed about the specifics of each stage just before they played it and were therefore blind to the order of the tasks. Using a one-shot PGG allows us to rule out the possibility that prosocial punishment was aimed at fostering future cooperation. Similarly, this setup prevents the emergence of reputational concerns or the development of social norms within the group. In other words, the one-shot PGG helps us better isolate the causal mechanism of interest by minimizing the influence of other potential factors that might otherwise be triggered. Further, by manipulating the presence of a redistribution stage, we can assess whether a redistributive institutional setting reduces the occurrence of prosocial punishment, decreasing waste of resources.

## Materials and methods

### Participants and procedure

To investigate the relationship between inequality, redistribution, and punishment, we ran a laboratory interactive game at the Laboratoire d’Economie Expérimentale de Nice (LEEN – Nice Lab, data collection from 06/04/2021 to 09/06/2021). This study was approved by the Ethics Committee of the School of Social Sciences of the Vrije Universiteit Amsterdam (RERC/2020-01-20, dated 20/01/2020). Informed consent was obtained from all participants through oTree; participants had to provide their consent before moving to the instruction page. The study involved 320 participants (*M*_*age*_ = 24.49, SD = 7.27, 101 males) across 80 groups. The experiment was programmed in oTree, version 3.4.0. Data and pre-registration files are available on the Open Science Framework website (https://osf.io/tq5gz).

We manipulate two factors in the experiment: the distribution of endowments among participants at the beginning of the game and the redistribution rule applied after the Public Goods Game (PGG) is completed. Thus, the experiment is a 2 (endowment inequality vs no inequality) X 2 (redistribution vs no redistribution) between-subjects design, see Table 1.

**Table 1:** Experimental treatments.

		Redistribution (R)	
		No R	R
Endowment	No EI	NEI&NR	NEI&R
Inequality (EI)	EI	EI&NR	EI&R

**Fig 1:**
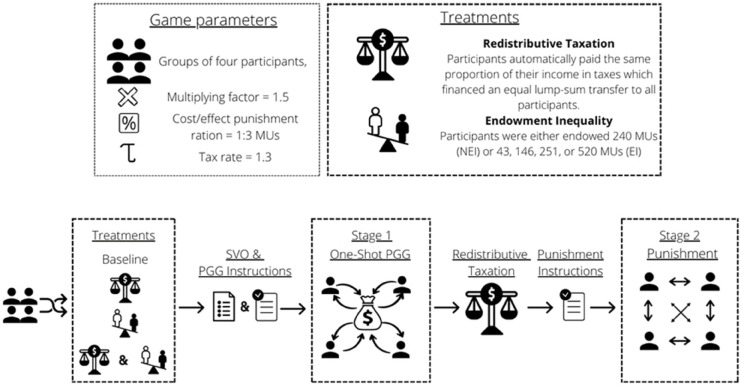
Experimental design. Participants were first arranged in groups of four and the groups were randomly assigned to a treatment. After, participants filled in the SVO questionnaire and were informed that the experiment consisted of two stages. They first read the instructions for stage 1, the Public Good Game (PGG). Participants were then assigned an initial endowment of (a) 240 MUs if they played in the NEI treatment, or (b) 43, 146, 251, or 520 MUs if they played in the EI treatment. They played a one-shot PGG after which they were informed of the other group members’ decisions and their payoff. Participants in NEI&R and EI&R were then informed that they were randomly allocated to a scenario where taxation was applied. Participants were explained the rule of taxation and were shown their own and the other group members’ payoff after taxation was applied. Finally, all participants read the instructions for stage 2, the punishment stage. Participants were endowed an additional 60 MUs and they could decide how many of these MUs to assign to each of their other group members, knowing that for each MU assigned, the payoff of the punished individual would decrease by 3 MUs.

First, participants completed the Social Value Orientation (SVO) task [21], which is our measure of inequality aversion preferences. Then, they played a one-shot PGG, followed by a redistribution stage (for participants in R), and a punishment stage. Finally, participants received a short questionnaire to gather sociodemographic information (see Fig 1 for more details).

Each group was randomly assigned to one of the above treatments and consisted of a planned number of 4 participants. Participants were informed about the structure of the game. In addition, they were told about the specifics of each stage just before they played it (not at the beginning of the experiment).

In the PGG, participants were informed of the endowment distribution at the beginning of the round. Different levels of inequality were calculated employing the following levels of Gini coefficients: 0.0 (no inequality); 0.4 (high inequality; in the most unequal countries in the world, such as the United States and Chile, the Gini-coefficient is around .4 [45]). Participants in NEI were endowed 240 monetary units (MU), while participants in EI were endowed 43, 146, 251, or 520 monetary units. Participants could decide how much they would contribute to the public good, knowing that the total resources would be multiplied by 1.5 and redistributed equally between all members of the group, regardless of their contribution. Participants were then informed about the decisions of their fellow group members, the new distribution of resources, and their placement in the distribution.

The redistribution rule was based on the Meltzer and Richard’s model [20]. We followed previous experimental research [46,47] and implemented a simplified version of this rule. These simplifications meant that we excluded production and incentive effects of taxation. Since participants did not have to exert effort, but were simply endowed with a number of tokens, this did not have an impact on our game. The rule consisted of proportional taxation and an equal lump-sum transfer to all participants:

$$y_{i}=(1-\tau )x_{i}+\tau \bar{x},$$ (1)

where *y*_*i*_ is the number of MUs participant *i* had after redistribution, *x*_*i*_ is the number of MUs participant *i* had after playing the PGG, $\bar{x}$ is the average amount of MUs after playing the PGG, and τ=1.3 is the tax rate. This rule implied that all participants paid the same proportion of their MUs in taxes which financed an equal lump-sum transfer to all participants; hence, the higher the amount of post-PGG MUs, the larger the payment was in absolute terms.

The punishment stage followed the mechanism proposed by Fehr and Gächter [28]: participants were endowed with an additional 60 MUs and they could decide to assign such units to punish their fellow group members. A punishment decision was implemented by assigning between 0 and 60 units to the punished member. Each unit assigned costs the punished member 3${\;\;}^{*}$MUs and the punishing member 1${\;\;}^{*}$MUs. All punishment decisions were made simultaneously. Notice that in our setup, punishment could not be a tool to enforce cooperation over time since the game was one-shot, and neither could it be a way of deterring defection since the punishment stage was not known at the time the PGG was played (in contrast to the setup in [28]).

### Measures

*Prosocial punishment.* Our dependent variable is calculated as the punishment allocated to individuals who contributed less than the average contribution in the PGG (hence, punishment of defectors).

*Inequality aversion.* Inequality aversion was calculated using the secondary SVO slider items as “this set of items is explicitly designed to disentangle the prosocial motivations of joint maximization from inequality aversion” ([21], p. 772). From the four separate mean difference measures that can be obtained from the secondary items of the SVO, we calculated the index to capture Inequity Aversion (IA) [48]. This index is used to differentiate between individuals who, within the prosocial region, are more inclined towards inequality aversion rather than joint gain maximisation. The resulting inequality aversion index is calculated as follows: $IA=\frac{DIA}{DIA+DJG}$, where *DIA* is the mean difference from archetypical inequality aversion and *DJG* is the mean difference from archetypical joint gain maximization. The ranges between 0 (indicating perfect inequality aversion) and 1 (indicating perfect joint gain maximization) [48]. The measure was reversed so that higher values would indicate stronger inequality aversion.

In addition, we indirectly gauge inequality aversion by analyzing the punishment behavior in the PGG. Specifically, in our exploratory analysis, prosocial punishment targeting richer individuals is interpreted as prosocial punishment motivated by inequality aversion.

### Statistical analysis

We employed tobit regression models evaluating the association between inequality aversion and prosocial punishment. In line with the literature, we used a tobit model for censored data (prosocial punishment) which has been found to have better performance compared to estimates from OLS regressions [49]. This model also allows us to (1) control for factors within the design that can influence the relationship, such as participants’ own contribution; (2) adjust for baseline covariates potentially unevenly distributed among groups (e.g., gender, age, ethnicity); (3) account for groups’ clustering. We interacted the treatment dummy variables with our measure of inequality aversion preferences (InequalityAversion × *Treatment*). In such a way, we assess whether and how the slope of the inequality aversion coefficient changes between treatments. In addition, in exploratory analysis, we employ multilevel tobit regression models to investigate prosocial punishment decisions, appropriately accounting for data structure (punishment decisions nested in subjects, nested in groups). Results from a sensitivity power analysis, see SM, show that we were able to detect effects of *d* = .32 for H1 and ${f\;}^{2}=\mathrm{.044}$ for H2. These effect sizes can be considered between small and medium (since .20< Cohen’s *d* < .50, and .02 < Cohen’s *f*^2^ < .15), as such this study is sensitive to detect effects between small and medium.

## Results

**Fig 2:**
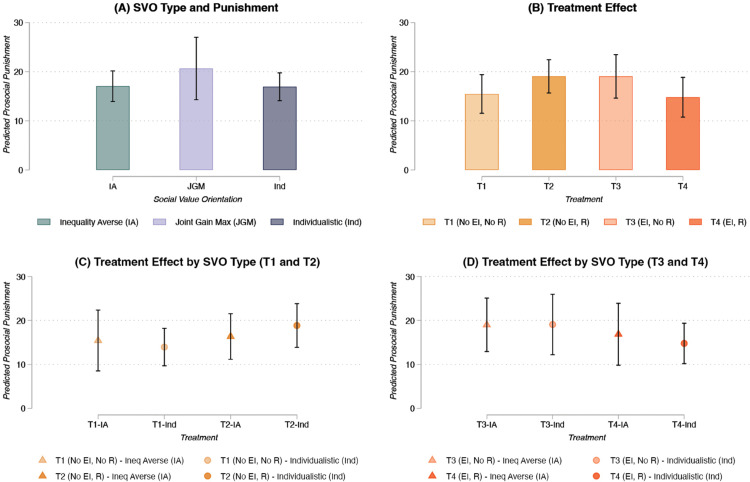
Prosocial punishment across SVO types and treatments. Panel A shows the predicted prosocial punishment values by SVO type with 95% CIs based on a tobit model (n=320) with SEs clustered at the group level (Table S3, Model 2). Panel B shows the predicted prosocial punishment values by treatment with 95% CIs based on a tobit model (n=320) with SEs clustered at the group level (Table S4, Model 2). Panels C-D show the predicted prosocial punishment values by SVO type (inequality averse, and individualistic) and treatment with 95% CIs based on a tobit model (n=320) with SEs clustered at the group level (Table S5, Model 2). Predicted values are based on the censored observed values. Controls include age, gender, ethnicity, PGG contribution, past lab participation, and number of known participants in the lab session. Results are robust to different modeling strategy and statistical controls (e.g., hurdle models, Table S7, and multilevel tobit models, Table S6). We set statistical significance at the 5% level (i.e., alpha=0.05) for two-sided tests.

Let us start by assessing the association between inequality aversion and prosocial punishment. The bivariate correlation between the inequality aversion index and prosocial punishment is negative and statistically significant (r = –.17, p < .05). However, this index considers only individuals with prosocial attitudes, as it takes lower values for individuals with stronger joint gain maximizing preferences and higher values for individuals with stronger inequality aversion (see Measures). When we consider more broadly the different types of preferences measured through the SVO (i.e., inequality averse, joint gain maximizing, and individualistic – altruistic is not reported since only one participant fell in this category), the association disappears: inequality aversion does not seem to significantly reduce the propensity to sanction free riders in our sample (see Fig 2, panel A). This evidence does not support our first pre-registered hypothesis.

Focusing on the effect of the different treatments (Fig 2, panel B), we can observe a null effect for both inequality and redistribution conditions, as well as for the combination of the two. Fig 2, Panels C and D illustrate that this is also the case for inequality averse individuals: applying a redistribution of resources before the punishment stage does not significantly change how inequality averse individuals sanction free riders compared to individuals with more individualistic preferences in conditions of no and high inequality. The same null results emerge when we compare inequality aversion with join gain maximizing preferences (see Supplementary Materials - SM, Table S5). These findings do not support H2a and H2b. Such results could also be connected to a measurement issue: the SVO may not fully capture the inequality aversion underlying the desire of sanction free riders, and therefore does not accurately predict prosocial punishment.

To further explore and better understand why prosocial punishment emerged in our experiment, we analyzed the data at the punishment decision level using multilevel tobit models, with each punishment decision nested in subjects who are nested in groups. We observe that our participants applied prosocial punishment in a way that is consistent with attitudinal measures of negative reciprocity (*B*_*NegRec*_=1.43, SE=.35 p < .001 - see SM, Table S9), in line with previous studies [50]. However, this does not mean that the punishment exerted may not have also been based (at least in part) on other motivations, such as inequality aversion. To assess whether this was the case, we analyzed how subjects punished richer individuals across treatments. Following the key idea behind our pre-registered hypotheses, we expected to see less punishment towards richer individuals when redistribution was applied. If punishment of defectors is due to inequality aversion (rather than negative reciprocity, or other related motivations), then the application of redistribution should significantly reduce sanctioning aimed at creating more equitable outcomes, as this is already largely addressed by the redistributive taxation. Punishment driven by negative reciprocity, on the other hand, should occur as usual since the redistribution of resources is implemented by a neutral third-party and it will not cancel out the unfair treatment received by the other participants (which should trigger sentiments of revenge and the consequent punishment).

**Fig 3:**
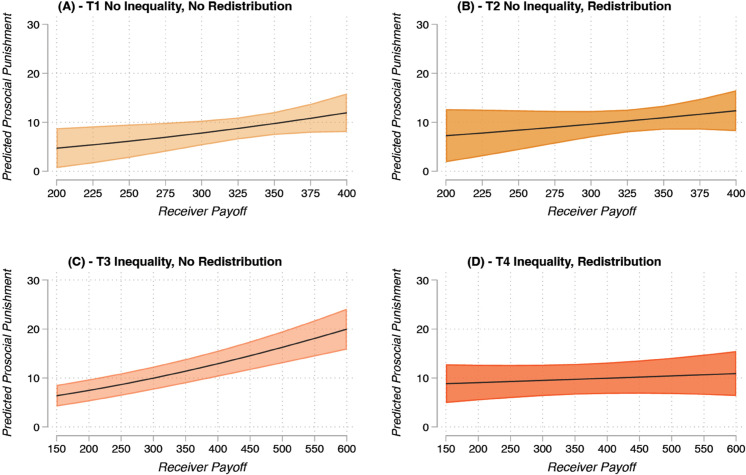
Prosocial punishment by treatment and receiver payoff. Panels A-D show the predicted prosocial punishment values by treatment and receiver payoff before the punishment stage with 95% CIs based on a multilevel tobit model with punishment decisions (n=543) nested in individuals (n=316) nested in groups (n=80, Table S8, Model 2). Predicted values are based on the censored observed values. Controls include age, gender, ethnicity, PGG contribution, past lab participation, and number of known participants in the lab session. Results are robust to different statistical controls (i.e., receiver PGG contribution - see Table S8, Model 3). We set statistical significance at the 5% level (i.e., alpha=0.05) for two-sided tests.

Fig 3 shows how participants punished defectors across treatments depending on their payoff before the start of the punishment stage. Comparing panels A and B, we can observe that there is no effect of redistribution in the case of no endowment inequality. However, there is a significant difference in how people punish when inequality is introduced exogenously: panel C indicates that richer individuals are punished more when there is no redistributive taxation (*AME*_*T*3_ = .031, SE = .005, p < .001), while panel D shows that, with redistributive taxation, richer individuals are punished as much as poorer individuals (*AME*_*T*4_ = .007, SE = .007, p = .302; Average Marginal Effects (AMEs) are computed using the censored observed values). The difference between the two slopes (i.e., the second difference) is statistically significant and suggests that, when inequality of endowments is high, redistribution does indeed reduce the intensity of punishment depending on the level of wealth of the receiver (*AME*_*T*4−*T*3_ = –.024, SE = .008, p = .003). Specifically, it indicates that an increase of 100 points on the receiver payoff leads to an average increase of 3.1 punishment points in T3, while in T4 the increase in punishment points would be statistically insignificant and equal to 0.7. This finding holds when controlling for the level of contribution of the punisher, negative reciprocity attitudes, and the level of contribution of the receiver (see SM, Table S8), suggesting that inequality aversion may indeed be a relevant driver of prosocial punishment when initial inequality is high.

## Discussion

Prosocial punishment has been extensively discussed as one of the key factors explaining cooperation among humans beyond close kin [11–13]. However, prosocial punishment is a costly behavior that can lead to resource wastage and possibly create friction within the social group [19,51,52]. Understanding why people engage in such behavior could help identify alternative ways to achieve the same goal (i.e., promoting cooperation) while reducing the risk of inefficiencies and social tensions. Although several studies have attempted to address this question, results have often been mixed (possibly due to the challenges of experimentally manipulating individual preferences) and point to a range of factors that may underlie prosocial peer punishment, including negative reciprocity [40,41], and inequality aversion [32,33]. In fact, when participants react to unequal outcomes with punishment, they may be responding to inequality itself, the norm violation that led to it, or simply reacting to the negative behavior of others.

In this study, we use a Public Goods Game (PGG) to investigate how the relationship between inequality aversion preferences (measured through the Social Value Orientation) and prosocial punishment is affected by inequality and redistribution. Specifically, we hypothesized that introducing a redistributive institutional setting before the punishment stage would make individuals motivated by inequality aversion less inclined to punish free riders, as their need for equality would have already been satisfied by the third-party redistribution system.

Our confirmatory analysis, however, shows null results for the relationship between stated inequality aversion preferences and prosocial peer punishment. In other words, individuals with higher inequality aversion (as measured through the SVO) did not punish free riders less under redistributive taxation. Because the SVO may not fully capture the specific inequality aversion motive underlying punitive behavior, these null results do not necessarily rule out inequality aversion as a driver of prosocial punishment. In line with our theoretical argument, we further investigated the relationship between inequality aversion and prosocial peer punishment. In our exploratory analysis, we observe that richer participants were punished more. This result relates to previous findings showing that endowment inequality in PGGs leads to less overall contributions [26], and that this is driven by the behavior of the rich [27]. Additionally, and more interestingly for our research question, we also find that redistributive taxation did affect punishing behaviors depending on others’ wealth when there was a high level of inequality in endowments. That is, the richer the participant, the more they were punished, and this pattern was mitigated by the introduction of redistributive taxation (see Fig 3, panels C and D). Previous findings suggest that the link between inequality and redistribution preferences may be affected by existing redistributive institutions, e.g., welfare regimes [53]. Our findings support this notion by showing that behavior likely motivated by inequality aversion is tempered by redistribution when high exogenous inequality is present.

From the point of view of resource wastage, we find that in T3 (inequality but no redistribution) poorer defectors are punished less since participants seem to allocate punishment not only based on defection, but also with an eye to inequality. A plausible interpretation is that punishers are balancing inequality aversion and negative reciprocity, drawing on both motives to some extent. Once the inequality aversion motive is satisfied, at least in part, by redistribution in T4, participants are free to act based on other motivations, such as negative reciprocity. An implication of this is that unchecked inequality may lead to under-provision of punishment in relation to defection since some (poor) defectors receive less punishment. More importantly, these results mean that our externally imposed “welfare regime” changes how punishment is allocated. With this redistributive rule in place, resources are not wasted on lowering the income of the rich but are focused on punishing defectors. Thus, redistribution matters for how inequality affects engagement in costly behavior.

These results have also implications for real-world expectations. For instance, our lab results may speak to previous research on how inequality changes attitudes or actions related to redistribution depending on whether the inequality arose fairly through effort or choices, or unfairly (e.g., cheating, luck, or inequality of opportunity) [54–57]. It also relates to previous research on how inequality in PGGs leads to lower contributions due to behavioral changes among wealthier participants [26,27]. At the same time, we should be cautious in generalising our results, as laboratory experiments often have limited external validity: while controlled conditions ensure internal rigour, they can also oversimplify or distort the social and economic dynamics present in natural settings [58,59].

Finally, our findings are directly relevant to the literature on the causes of peer punishment. In line with Bone and Raihani, 2015 [32], we find evidence suggesting that both negative reciprocity and inequality aversion play a role (but in a PGG setting rather than a moonlighting game). The observed behavior suggests that participants were not just concerned with the equal distribution of payoffs, since the average degree of punishment was not statistically different even in the presence of redistribution. It is also important to notice that, although our interpretation of the results focuses on inequality aversion and (more marginally) on negative reciprocity, this does not exclude the possibility that redistribution affects behavior through other channels, such as social comparison.

## Conclusion

By introducing an equalizing tax-transfer stage in the standard one-shot PGG with punishment, we were able to contribute to the debate about the underlying causes of prosocial punishment. By ruling out motives related to the enforcement of cooperation, we focused on inequality aversion. We found that even in the absence of opportunities to enforce cooperation and with exogenous redistribution, people are still willing to incur costs to reduce the payoff of norm violators: redistribution does not affect the overall level of punishment, but its distribution. This has relevance for real-world political behavior, such as voting for higher taxes on the rich. Scheidel [9] highlights that throughout history, a more equal distribution of wealth has been the result of violence. In a modern democratic welfare state, redistribution is peaceful.

The results show that redistribution reduces the need for prosocial punishment driven by inequality aversion by “correcting” for the initial inequality of endowments. Interestingly, we find that even when we account for inequality aversion as a motive – either by controlling for it with the SVO and by manipulating redistribution – there is residual prosocial punishment. Although our design cannot definitively identify its source, the leading explanation in the literature is negative reciprocity, and our findings are consistent with that interpretation.

However, our approach also has limitations. Alternative methods such as moonlighting games (used by [32]), or mini-dictator games could potentially capture who is motivated by inequality aversion in a more direct way. Another limitation is that in our simple setup, participants cannot influence taxes and transfers. Further research could investigate whether democratic control of redistribution matters (see [60]). Giving participants influence over the centrally managed redistributive policy would be a natural extension. Further research could also investigate whether participants prefer migrating to a tax-transfer institution, for instance if they would actively choose the tax treatment by migrating to PGG groups with this redistribution rule in place (see for example [15,61]). Our results lead us to suspect that even in these cases there would be positive levels of prosocial punishment (motivated by negative reciprocity); even in more equal societies, norm violators should expect consequences. Finally, future research in the area of inequality and PGGs (e.g., [26,27]) could investigate whether redistribution affects behavior in a similar way also with respect to contributions.

## Supporting information

S1 Text Supplementary Materials contain sensitivity power analysis, descriptive statistics, confirmatory and exploratory analysis full results tables and robustness checks, and experimental screenshots. (DOCX)
