# Supplementary material for: Inequality aversion and prosocial punishment: Evidence from a one-shot public goods game
Source: PLoS One. 2026 Jan 7;21(1):e0337425. doi: 10.1371/journal.pone.0337425 (PMC12779032; doi:10.1371/journal.pone.0337425)
Supplement: S1 Text — (DOCX) [file pone.0337425.s001.docx]

The Impact of Redistributive Taxation on Prosocial Punishment

Supplementary Materials

Table of Contents

[S1. Sensitivity Power Analyss 2](#_Toc209623403)

[S2. Descriptive statistics 3](#_Toc209623404)

[S3. Confirmatory analysis results and robustness checks 4](#_Toc209623405)

[S4. Exploratory analysis results and robustness checks 8](#_Toc209623406)

[S5. Experiment screenshots 12](#_Toc209623407)

[S5. References 19](#_Toc209623408)

# S1. Sensitivity Power Analysis

We conducted a sensitivity power analysis for the main models testing H1 (model 1 in Table S3 in the SM) and H2 (model 1 in Table S5 in the SM), investigating which effect sizes our sample could detect with 80% power (1 – β) and an alpha level of α = .05.

For model 1 in Table S3, since our first hypothesis suggests that individuals who fall in the inequality averse category will exercise more punishment than individuals falling in other categories, we used the “t-tests” test family with the statistical test “Means: Difference between two independent means (two groups)”. We input .05 for “α err prob”, 0.8 for “power (1 – β err prob)”, two-tailed test, and the sample size for each group. Following recommendations for power analyses for interactions (Perugini, Gallucci, & Costantini, 2018) for model 1 in Table S5, we used the “F-tests” test family with the statistical test “Linear multiple regression: Fixed model, R2 increase”. We input 6 for “number of tested predictors” (the interaction terms) and 12 “total number of predictors” (the interactions and the main effects). We further input .05 for “α err prob”, 0.8 for “power (1 – β err prob)”, and the sample size for each model respectively for the “total sample size”. We interpret the Cohen’s *d* and *f*^2^ according to rules of thumb (Cohen, 2013) as indicated in G*power (Perugini, Gallucci, & Costantini, 2018).

Results indicate that we were able to detect effects of *d* = .32 for H1 and *f^2^* = .044 for H2. These effect sizes can be considered between small and medium (since .20 < Cohen’s *d* < .50, and .02 < Cohen’s *f^2^* < .15), as such this study is sensitive to detect effects between small and medium.

# S2. Descriptive statistics

The experiment was conducted between April and June 2021 in the Laboratoire d’Economie Experimentale de Nice (LEEN– Nice Lab). The experiment was programmed in oTree version 3.4.0.

Participants were paid a reward of €5 as a showing up fee. In addition, they earned monetary bonuses based on their decisions during the experiment. Participants earned on average €4.16 in bonuses (SD=1.00, max=€7.87), excluding the showing up fee.

**Table S1**.Descriptive statistics.

|  | mean | sd | min | max | count |
| --- | --- | --- | --- | --- | --- |
| Gender |  |  |  |  |  |
| Male | 0.32 | 0.47 | 0 | 1 | 320 |
| Female | 0.68 | 0.47 | 0 | 1 | 320 |
| Other | 0.01 | 0.08 | 0 | 1 | 320 |
| Age | 24.49 | 7.27 | 18 | 71 | 320 |
| Number of known participants in the lab session | 0.45 | 0.94 | 0 | 10 | 320 |
| Past lab participation | 1.02 | 1.21 | 0 | 7 | 320 |
| Participant contribution in the PGG | 39.94 | 32.41 | 0 | 100 | 320 |
| Ethnicity (Minority yes or no) | | |  |  |  |
| Minority | 0.28 | 0.45 | 0 | 1 | 320 |
| SVO main type | |  |  |  |  |
| Altruistic | 0 | 0.06 | 0 | 1 | 320 |
| Prosocial | 0.58 | 0.49 | 0 | 1 | 320 |
| Individualistic | 0.41 | 0.49 | 0 | 1 | 320 |
| Competitive | 0.01 | 0.08 | 0 | 1 | 320 |
| SVO secondary type | |  |  |  |  |
| Inequality aversion | 0.4 | 0.49 | 0 | 1 | 320 |
| Joint gain maximiser | 0.13 | 0.33 | 0 | 1 | 320 |
| Individualistic | 0.47 | 0.5 | 0 | 1 | 320 |
| Altruistic | 0 | 0.06 | 0 | 1 | 320 |
| Treatments |  |  |  |  |  |
| NEI&NR | 0.28 | 0.45 | 0 | 1 | 320 |
| NEI&R | 0.3 | 0.46 | 0 | 1 | 320 |
| EI&NR | 0.28 | 0.45 | 0 | 1 | 320 |
| EI&R | 0.15 | 0.36 | 0 | 1 | 320 |
| Prosocial punishment | 17.33 | 20.43 | 0 | 60 | 320 |

**Note**. NEI&NR: No endowment inequality & no redistribution; NEI&R: No endowment inequality & redistribution; EI&NR: Endowment inequality & no redistribution; EI&R: Endowment inequality & redistribution.

# S3. Confirmatory analysis results and robustness checks

**Table S2**. Tobit model estimating the level of prosocial punishment by treatment and inequality aversion index with and without demographic controls.

| DV: Prosocial Punishment | Model 1 | Model 2 | Model 3 |
| --- | --- | --- | --- |
| Inequality Aversion index (IA) | -24.05*  (11.23) | -23.878*  (11.12) | -30.945  (23.06) |
| *Gender (ref: male)* | | | |
| Female |  | 10.438  (6.422) | 9.777  (7.074) |
| Other |  | 29.227*  (11.203) | 26.742*  (11.786) |
| Age |  | -0.567  (0.498) | -0.600  (0.515) |
| *Ethnicity (ref: non minority)* | | | |
| Minority |  | 1.656  (5.507) | 1.589  (5.766) |
| Participant contribution in the PGG |  | 0.089  (0.091) | 0.098  (0.094) |
| Number of known participants in the lab session |  | 1.348  (1.578) | 1.363  (1.628) |
| Past lab participation |  | -0.634  (1.713) | -0.653  (1.847) |
| *Treatments (ref: NEI&NR)* | | | |
| NEI&R |  |  | -1.742  (20.145) |
| EI&NR |  |  | -10.476  (18.924) |
| EI&R |  |  | -20.278  (34.576) |
| *Interaction terms* | | | |
| NEI&R x IA |  |  | 3.355  (30.002) |
| EI&NR x IA |  |  | 16.702  (28.138) |
| EI&R x IA |  |  | 24.399  (48.803) |
| Constant | 27.736***  (7.685) | 29.017*  (14.596) | 34.766  (19.188) |
| N | 145 | 145 | 145 |

**Note.** Standard errors clustered at the group level in parentheses. Significance levels: * p<0.05, ** p<0.01, *** p<0.001. NEI&NR: No endowment inequality & no redistribution; NEI&R: No endowment inequality & redistribution; EI&NR: Endowment inequality & no redistribution; EI&R: Endowment inequality & redistribution.

**Table S3**. Tobit model estimating the level of prosocial punishment by SVO type with and without demographic controls.

| DV: Prosocial Punishment | Model 1 | | Model 2 |
| --- | --- | --- | --- |
| *SVO type (ref: Inequality aversion)* |  | |  |
| Joint gain maximiser | 5.270  (5.722) | | 5.395  (5.295) |
| Individualistic | -2.107  (3.465) | | -0.194  (3.414) |
| Altruistic | -140.631***  (7.932) | | -139.137***  (8.619) |
| *Gender (ref: male)* | |  |  |
| Female |  | | 10.118*  (3.931) |
| Other |  | | 30.360***  (9.127) |
| Age |  | | -0.170  (0.265) |
| *Ethnicity (ref: non minority)* | |  |  |
| Minority |  | | \| 0.620  (3.359) \| \| --- \| |
| Participant contribution in the PGG |  | | 0.189***  (0.056) |
| Number of known participants in the lab session |  | | 0.715  (1.451) |
| Past lab participation |  | | -0.377  (1.257) |
| Constant | 10.652***  (2.682) | | -0.684  (8.826) |
| N | 320 | | 320 |

**Note.** Only one participant scored altruistic in the SVO type, coefficients for altruistic should be disregarded. Standard errors clustered at the group level in parentheses. Significance levels: * p<0.05, ** p<0.01, *** p<0.001. NEI&NR: No endowment inequality & no redistribution; NEI&R: No endowment inequality & redistribution; EI&NR: Endowment inequality & no redistribution; EI&R: Endowment inequality & redistribution.

**Table S4**.Tobit model estimating the level of prosocial punishment by treatment with and without demographic controls.

| DV: Prosocial Punishment | Model 1 | | Model 2 |
| --- | --- | --- | --- |
| *Treatments (ref: EI&NR)* |  | |  |
| NEI&NR | -4.562 | | -5.630 |
|  | (4.705) | | (4.750) |
| NEI&R | -0.429 | | 0.000 |
|  | (4.266) | | (4.274) |
| EI&R | -6.131 | | -6.753 |
|  | (4.481) | | (4.914) |
| *Gender (ref: male)* | |  |  |
| Female |  | | 9.955* |
|  |  | | (3.853) |
| Other |  | | 32.571** |
|  |  | | (12.245) |
| Age |  | | -0.193 |
|  |  | | (0.254) |
| *Ethnicity (ref: non minority)* | |  |  |
| Minority |  | | 0.764 |
|  |  | | (3.242) |
| Participant contribution in the PGG |  | | 0.197*** |
|  |  | | (0.055) |
| Number of known participants in the lab session |  | | 0.901 |
|  |  | | (1.451) |
| Past lab participation |  | | -0.221 |
|  |  | | (1.268) |
| Constant | 12.529*** | | 2.415 |
|  | (3.655) | | (8.711) |
| N | 320 | | 320 |

**Note.** Standard errors clustered at the group level in parentheses. Significance levels: * p<0.05, ** p<0.01, *** p<0.001. NEI&NR: No endowment inequality & no redistribution; NEI&R: No endowment inequality & redistribution; EI&NR: Endowment inequality & no redistribution; EI&R: Endowment inequality & redistribution.

**Table S5**.Tobit model estimating the level of prosocial punishment by treatment and SVO type with and without demographic controls.

| DV: Prosocial Punishment | Model 1 | | Model 2 |
| --- | --- | --- | --- |
| *Treatments (ref: EI&NR)* |  | |  |
| NEI&NR | -7.653 | | -6.779 |
|  | (7.173) | | (7.645) |
| NEI&R | -3.711 | | -3.639 |
|  | (6.419) | | (6.218) |
| EI&R | -4.769 | | -4.115 |
|  | (6.297) | | (7.283) |
| *SVO type (ref: Inequality aversion)* |  | |  |
| Joint gain maximiser (JGM) | -1.665 | | -0.009 |
|  | (9.731) | | (8.677) |
| Individualistic (I) | -3.015 | | 0.094 |
|  | (6.441) | | (6.489) |
| Altruistic (A) | -143.112*** | | -140.591*** |
|  | (8.789) | | (9.830) |
| *Interaction terms* |  | |  |
| NEI&NR x JGM | 17.584 | | 12.931 |
|  | (14.657) | | (13.070) |
| NEI&NR x I | 0.180 | | -2.656 |
|  | (9.483) | | (9.630) |
| NEI&R x JGM | 10.775 | | 10.688 |
|  | (13.482) | | (12.406) |
| NEI&R x I | 3.578 | | 3.799 |
|  | (9.522) | | (8.862) |
| EI&R x JGM | -22.097 | | -19.916 |
|  | (22.922) | | (20.930) |
| EI&R x I | -0.415 | | -3.509 |
|  | (8.919) | | (8.961) |
| *Gender (ref: male)* | |  |  |
| Female |  | | 10.714** |
|  |  | | (4.076) |
| Other |  | | 28.769*** |
|  |  | | (8.536) |
| Age |  | | -0.180 |
|  |  | | (0.253) |
| Number of known participants in the lab session |  | | 0.824 |
|  |  | | (1.495) |
| Participant contribution in the PGG |  | | -0.368 |
|  |  | | (1.272) |
| *Ethnicity (ref: non minority)* | |  |  |
| Minority |  | | 0.731 |
|  |  | | (3.271) |
| Past lab participation |  | | 0.187*** |
|  |  | | (0.055) |
| Constant | 14.536** | | 2.605 |
|  | (4.387) | | (9.601) |
| N | 320 | | 320 |

**Note.** Only one participant scored altruistic in the SVO type, coefficient for altruistic should be disregarded. The participant scoring altruistic in the SVO type was in EI&RN treatment, thus the interaction between altruistic and the other treatments is empty. Standard errors clustered at the group level in parentheses. Significance levels: * p<0.05, ** p<0.01, *** p<0.001. NEI&NR: No endowment inequality & no redistribution; NEI&R: No endowment inequality & redistribution; EI&NR: Endowment inequality & no redistribution; EI&R: Endowment inequality & redistribution.

# S4. Exploratory analysis results and robustness checks

**Table S6**. Multilevel tobit model estimating the level of prosocial punishment by treatment and SVO type with and without demographic controls.

| DV: Prosocial Punishment | Model 1 | | Model 2 | | Model 3 |
| --- | --- | --- | --- | --- | --- |
| *Treatments (ref: NEI&NR)* |  | |  | |  |
| NEI&R |  | | 5.634 | | 3.139 |
|  |  | | (4.365) | | (7.063) |
| EI&NR |  | | 6.087 | | 6.778 |
|  |  | | (4.437) | | (6.992) |
| EI&R |  | | -1.153 | | 2.663 |
|  |  | | (5.298) | | (8.171) |
| *SVO type (ref: Inequality aversion)* |  | |  | |  |
| Joint gain maximiser | 5.395 | |  | | 12.922 |
|  | (5.318) | |  | | (9.536) |
| Individualistic | -0.194 | |  | | -2.561 |
|  | (3.604) | |  | | (6.931) |
| *Interaction terms* |  | |  | |  |
| NEI&R x JGM |  | |  | | -2.243 |
|  |  | |  | | (13.687) |
| EI&NR x JGM |  | |  | | -12.931 |
|  |  | |  | | (13.282) |
| EI&R x JGM |  | |  | | -32.845 |
|  |  | |  | | (20.805) |
| NEI&R x I |  | |  | | 6.454 |
|  |  | |  | | (9.467) |
| EI&NR x I |  | |  | | 2.656 |
|  |  | |  | | (9.842) |
| EI&R x I |  | |  | | -0.853 |
|  |  | |  | | (11.224) |
| *Gender (ref: male)* | |  | |  |  |
| Female | 10.117** | | 9.554** | | 10.713** |
|  | (3.718) | | (3.652) | | (3.762) |
| Other | 30.358 | | 32.044 | | 28.768 |
|  | (20.002) | | (19.878) | | (20.135) |
| Age | -0.170 | | -0.199 | | -0.180 |
|  | (0.229) | | (0.227) | | (0.228) |
| Number of known participants in the lab session | 0.715 | | 0.875 | | 0.824 |
|  | (1.801) | | (1.781) | | (1.814) |
| Past lab participation | -0.377 | | -0.286 | | -0.368 |
|  | (1.395) | | (1.383) | | (1.394) |
| *Ethnicity (ref: non minority)* | |  | |  |  |
| Minority | 0.619 | | 0.609 | | 0.731 |
|  | (3.727) | | (3.705) | | (3.710) |
| Participant contribution in the PGG | 0.189*** | | 0.203*** | | 0.187*** |
|  | (0.052) | | (0.052) | | (0.053) |
| Constant | -0.682 | | -2.880 | | -4.172 |
|  | (7.514) | | (7.619) | | (8.628) |
| Individuals/Groups | 319/80 | | 319/80 | | 319/80 |

**Note.** In order for the model to converge, we dropped the one individual who scored altruistic in the SVO type. Significance levels: * p<0.05, ** p<0.01, *** p<0.001. NEI&NR: No endowment inequality & no redistribution; NEI&R: No endowment inequality & redistribution; EI&NR: Endowment inequality & no redistribution; EI&R: Endowment inequality & redistribution.

**Table S7**. Hurdle model estimating the level of prosocial punishment by treatment and SVO type with and without demographic controls.

| DV: Prosocial Punishment | Model 1  First Hurdle | Model 1  Second Hurdle | Model 2  First Hurdle | Model 2  Second Hurdle |  |
| --- | --- | --- | --- | --- | --- |
| *Treatments (ref: NEI&NR)* |  |  |  |  |  |
| NEI&R | 2.054 | 0.277 | -3.067 | 0.224 |  |
|  | (6.111) | (0.175) | (11.038) | (0.272) |  |
| EI&NR | 4.167 | 0.243 | -3.549 | 0.414 |  |
|  | (5.161) | (0.214) | (9.015) | (0.325) |  |
| EI&R | -12.588 | 0.209 | -11.913 | 0.395 |  |
|  | (9.131) | (0.210) | (10.835) | (0.349) |  |
| *SVO type (ref: Inequality aversion)* |  |  |  |  |  |
| Joint gain maximiser (JGM) | -3.737 | 0.320 | -6.192 | 0.748* |  |
|  | (8.278) | (0.231) | (15.741) | (0.341) |  |
| Individualistic (I) | -4.989 | 0.037 | -12.909 | 0.056 |  |
|  | (5.978) | (0.144) | (12.678) | (0.244) |  |
| Altruistic | 0 | -5.109*** | 0 | -4.736*** |  |
|  | (.) | (0.360) | (.) | (0.425) |  |
| *Interaction terms* |  |  |  |  |  |
| JGM x NEI&R |  |  | 4.743 | -0.096 |  |
|  |  |  | (21.370) | (0.611) |  |
| JGM x EI&NR |  |  | 1.910 | -0.579 |  |
|  |  |  | (21.236) | (0.542) |  |
| JGM x EI&R |  |  | 35.883 | -1.913** |  |
|  |  |  | (19.213) | (0.715) |  |
| I x NEI&R |  |  | 9.964 | 0.133 |  |
|  |  |  | (18.216) | (0.367) |  |
| I x EI&NR |  |  | 18.264 | -0.220 |  |
|  |  |  | (15.248) | (0.414) |  |
| I x EI&R |  |  | -3.774 | -0.007 |  |
|  |  |  | (20.646) | (0.467) |  |
| *Gender (ref: male)* |  |  |  | |  |
| Female | -1.723 | 0.583*** | -0.125 | 0.598*** |  |
|  | (5.367) | (0.155) | (5.785) | (0.161) |  |
| Other | 4.405 | 5.109*** | 5.790 | 4.675*** |  |
|  | (12.558) | (0.208) | (13.706) | (0.277) |  |
| Age | -0.360 | 0.002 | -0.311 | 0.001 |  |
|  | (0.473) | (0.011) | (0.447) | (0.011) |  |
| Number of known participants in the lab session | -3.819 | 0.136 | -3.253 | 0.122 |  |
|  | (3.141) | (0.084) | (3.079) | (0.085) |  |
| Past lab participation | 0.580 | -0.042 | 0.052 | -0.041 |  |
|  | (2.001) | (0.066) | (2.009) | (0.068) |  |
| *Ethnicity (ref: non minority)* |  |  |  | |  |
| Minority | -7.143 | 0.171 | -6.914 | 0.171 |  |
|  | (5.479) | (0.172) | (5.477) | (0.176) |  |
| Participant contribution in the PGG | 0.403*** | 0.002 | 0.398*** | 0.002 |  |
|  | (0.072) | (0.002) | (0.072) | (0.002) |  |
| Constant | 13.616 | -0.451 | 16.015 | -0.490 |  |
|  | (14.532) | (0.373) | (14.516) | (0.396) |  |
| N | 320 | | 320 | |  |

**Note.** Standard errors clustered at the group level in parentheses. Only one participant scored altruistic in the SVO type, coefficient for altruistic should be disregarded. The participant scoring altruistic in the SVO type was in EI&RN treatment, thus the interaction between altruistic and the other treatments is empty. Significance levels: * p<0.05, ** p<0.01, *** p<0.001. NEI&NR: No endowment inequality & no redistribution; NEI&R: No endowment inequality & redistribution; EI&NR: Endowment inequality & no redistribution; EI&R: Endowment inequality & redistribution.

**Table S8**. Multilevel tobit model estimating the level of prosocial punishment by treatment and receiver payoff with and without demographic controls.

| DV: Prosocial Punishment | Model 1 | | Model 2 | | Model 3 |
| --- | --- | --- | --- | --- | --- |
| *Treatments (ref:EI&NR)* |  | |  | |  |
| NEI&NR | -18.009 | | -19.630 | | -19.630 |
|  | (13.734) | | (13.877) | | (13.877) |
| NEI&R | -14.486 | | -16.557 | | -16.557 |
|  | (13.375) | | (13.469) | | (13.469) |
| EI&R | 11.956* | | 13.088* | | 13.088* |
|  | (6.076) | | (6.036) | | (6.036) |
| Receiver payoff | 0.051*** | | 0.052*** | | 0.052*** |
|  | (0.007) | | (0.007) | | (0.007) |
| Receiver contribution |  | |  | | -0.196*** |
|  |  | |  | | (0.058) |
| *Interaction terms* |  | |  | |  |
| NEI&NR x receiver payoff | 0.045 | | 0.049 | | 0.023 |
|  | (0.040) | | (0.040) | | (0.040) |
| NEI&R x receiver payoff | 0.045 | | 0.051 | | 0.004 |
|  | (0.039) | | (0.039) | | (0.041) |
| EI&R x receiver payoff | -0.039** | | -0.040** | | -0.041** |
|  | (0.013) | | (0.013) | | (0.013) |
| *Gender (ref: male)* | |  | |  |  |
| Female |  | | 8.257*** | | 8.244*** |
|  |  | | (2.434) | | (2.400) |
| Other |  | | 25.432 | | 23.666 |
|  |  | | (13.308) | | (13.120) |
| Age |  | | -0.110 | | -0.131 |
|  |  | | 8.257*** | | (0.149) |
| Number of known participants in the lab session |  | | (0.151) | | 0.161 |
|  |  | | 0.401 | | (1.159) |
| Past lab participation |  | | (1.174) | | -0.316 |
|  |  | | -0.198 | | (0.908) |
| *Ethnicity (ref: non minority)* | |  | |  |  |
| Minority |  | | 1.466 | | 1.445 |
|  |  | | (2.459) | | (2.424) |
| Participant contribution in the PGG |  | | -0.011 | | 0.034 |
|  |  | | (0.036) | | (0.037) |
| Constant | -11.889*** | | -15.313** | | -10.886 |
|  | (3.366) | | (5.638) | | (5.686) |
| Punishment decisions/Individuals/Groups | 543/316/80 | | 543/316/80 | | 543/316/80 |

**Note.** Significance levels: * p<0.05, ** p<0.01, *** p<0.001. NEI&NR: No endowment inequality & no redistribution; NEI&R: No endowment inequality & redistribution; EI&NR: Endowment inequality & no redistribution; EI&R: Endowment inequality & redistribution.

**Table S9**. Multilevel tobit model estimating the level of prosocial punishment by negative reciprocity with and without controls.

| DV: Prosocial Punishment | Model 1 | | Model 2 | |
| --- | --- | --- | --- | --- |
| Negative Reciprocity | 1.429*** (0.345) | | 1.530***  (0.343) | |
| *Treatments (ref:EI&NR)* |  | |  | |
| NEI&NR |  | | 3.868 | |
|  |  | | (2.794) | |
| NEI&R |  | | 4.834 | |
|  |  | | (2.838) | |
| EI&R |  | | 2.116 | |
|  |  | | (3.470) | |
| *SVO type (ref: Inequality aversion)* |  | |  | |
| Joint gain maximiser (JGM) |  | | 3.940 | |
|  |  | | (3.410) | |
| Individualistic (I) |  | | -1.173 | |
|  |  | | (2.316) | |
| Altruistic |  | | -108.493 | |
|  |  | | (109975.808) | |
| *Gender (ref: male)* | |  | |  |
| Female |  | | 8.135*** | |
|  |  | | (2.383) | |
| Other |  | | 29.580* | |
|  |  | | (12.961) | |
| Age |  | | -0.068 | |
|  |  | | (0.145) | |
| Number of known participants in the lab session |  | | -0.022 | |
|  |  | | (1.134) | |
| Past lab participation |  | | 0.141 | |
|  |  | | (0.895) | |
| *Ethnicity (ref: non minority)* | |  | |  |
| Minority |  | | 1.592 | |
|  |  | | (2.385) | |
| Participant contribution in the PGG |  | | 0.028 | |
|  |  | | (0.034) | |
| Constant | -2.003 | | -10.949* | |
|  | (2.024) | | (5.446) | |
| Punishment decisions/Individuals/Groups | 543/316/80 | | 543/316/80 | |

**Note.** Significance levels: * p<0.05, ** p<0.01, *** p<0.001. NEI&NR: No endowment inequality & no redistribution; NEI&R: No endowment inequality & redistribution; EI&NR: Endowment inequality & no redistribution; EI&R: Endowment inequality & redistribution.

# S5. Experiment screenshots

#
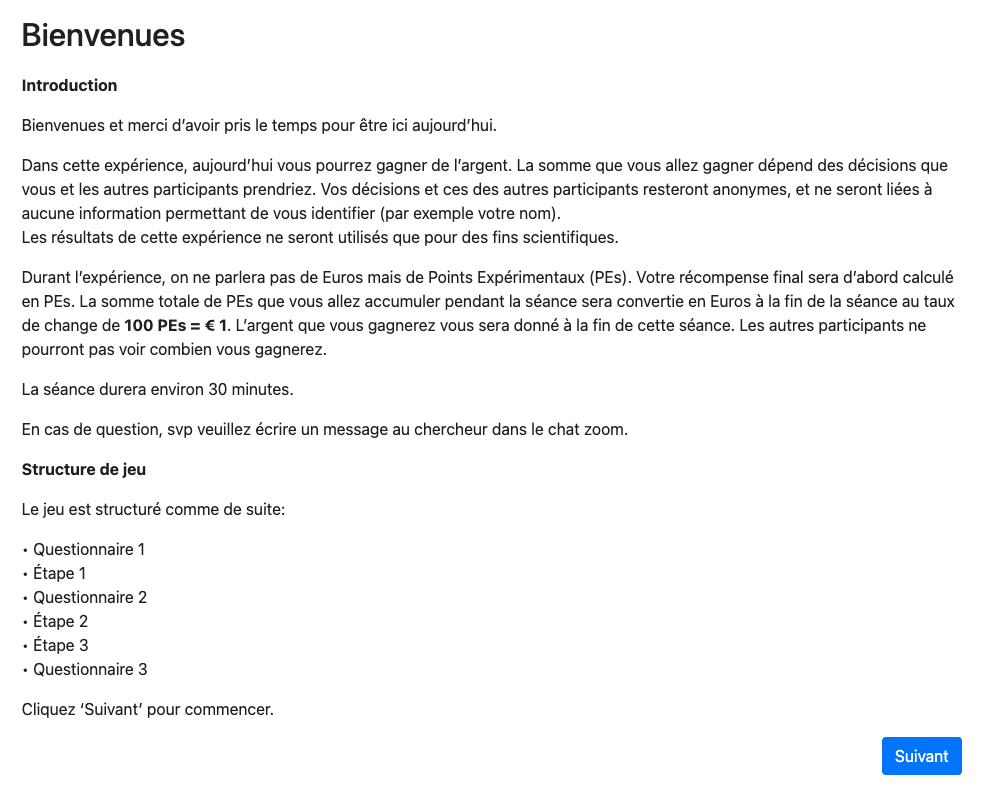


**Figure S1.** Welcome Page


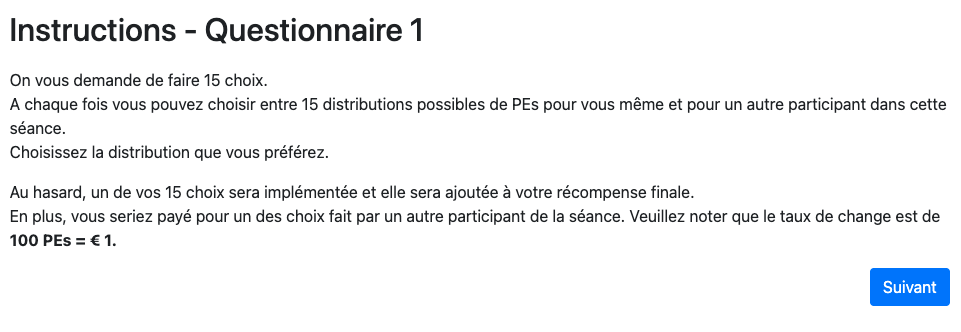


**Figure S2.** Instructions Social Value Orientation


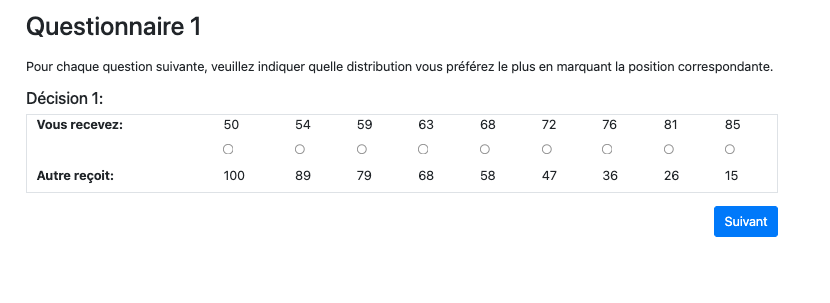
**Figure S3.** Social Value Orientation Decisions


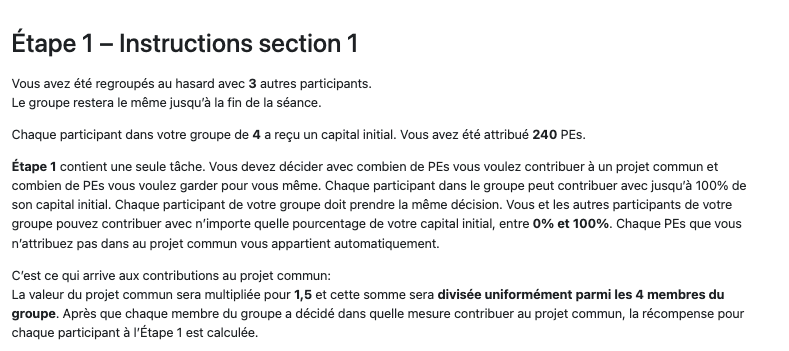


**Figure S4.** Step 1, Instructions Public Goods Game


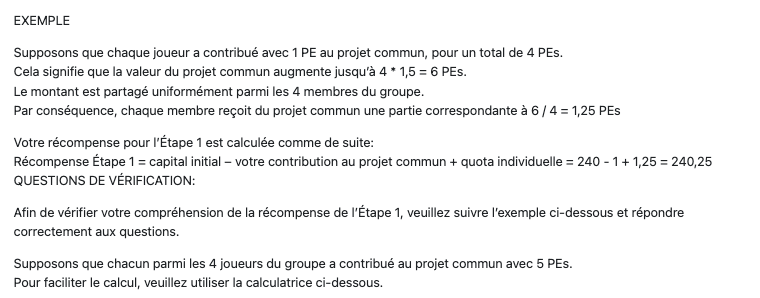


**Figure S5.** Step 1, Example Public Goods Game


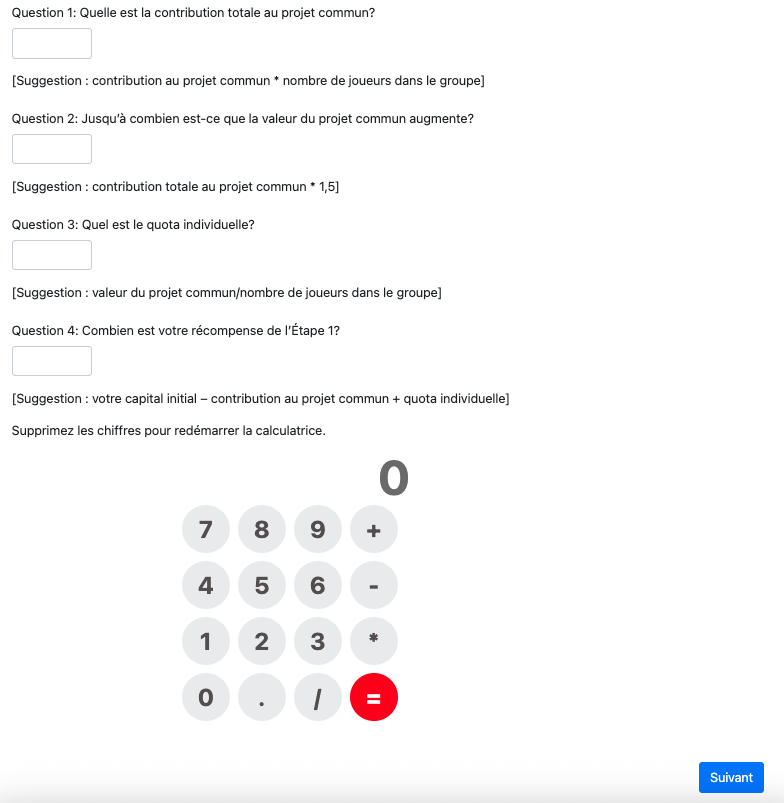


**Figure S6.** Comprehension Questions Public Goods Game


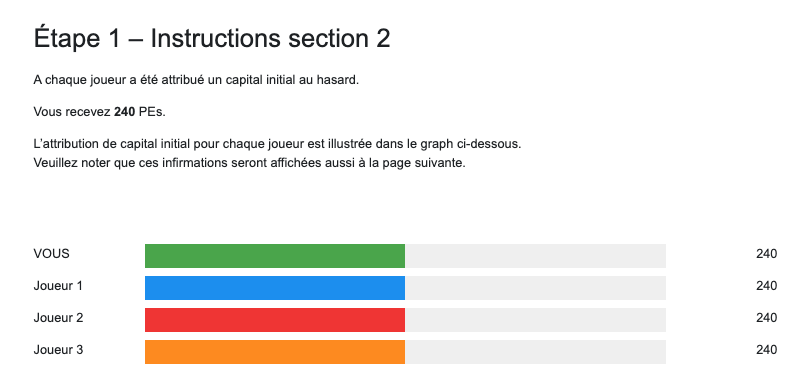


**Figure S7.** Step 2, Instructions Public Goods Game, No Inequality


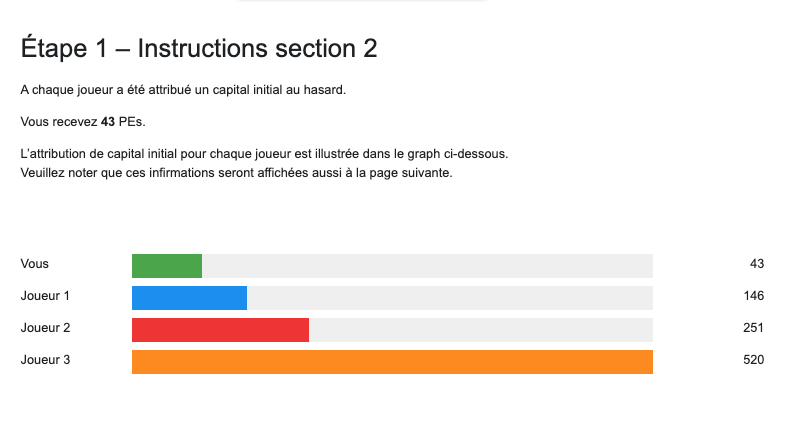


**Figure S8.** Step 2, Instructions Public Goods Game, Inequality


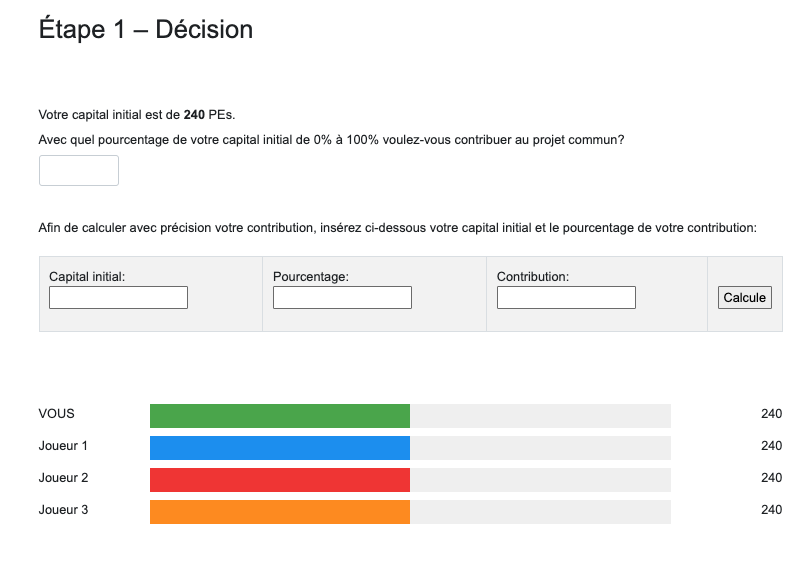


**Figure S9.** Public Goods Game Contribution, No Inequality


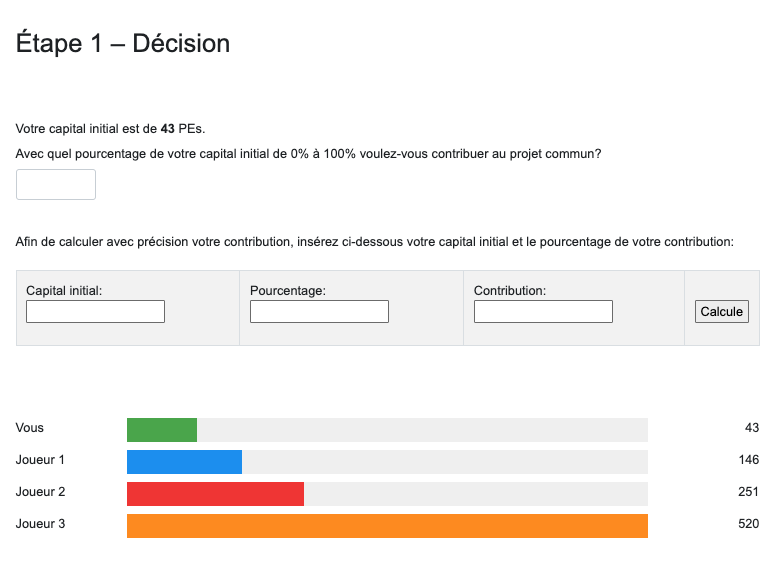


**Figure S10.** Public Goods Game Contribution, Inequality


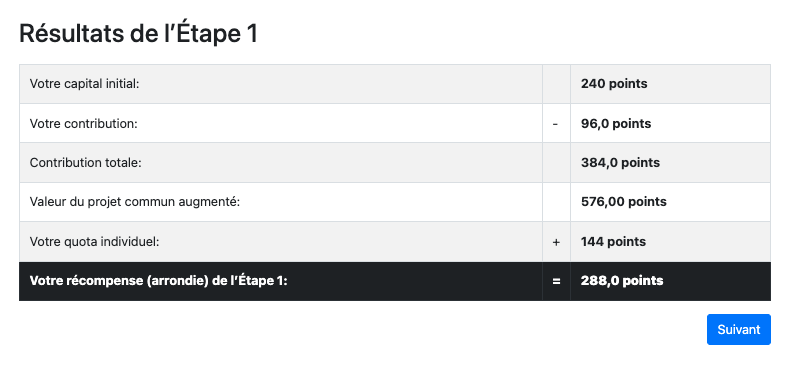


**Figure S11.** Public Goods Game Results


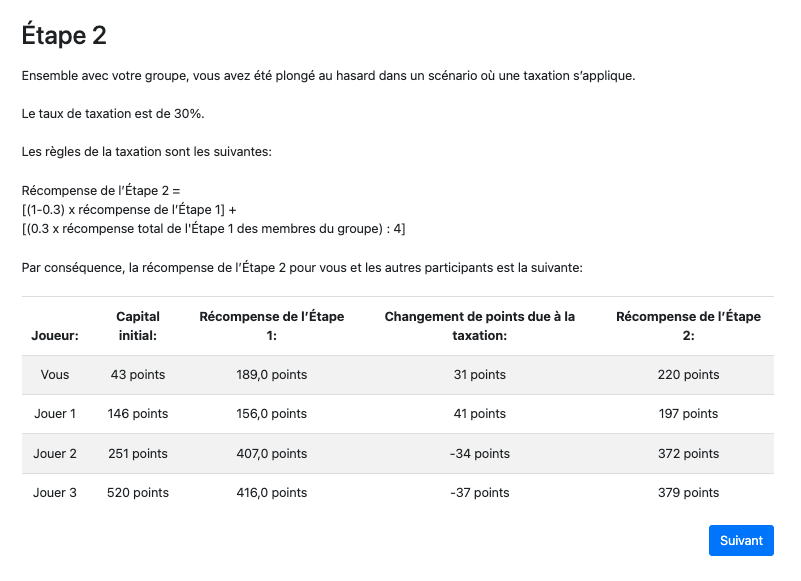


**Figure S12.** Step 2, Redistribution


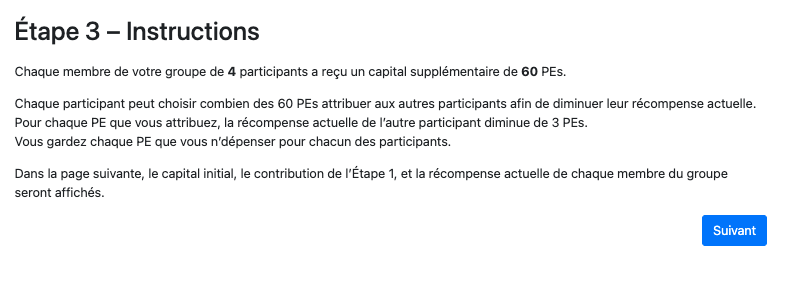


**Figure S13.** Step 3, Punishment Instructions


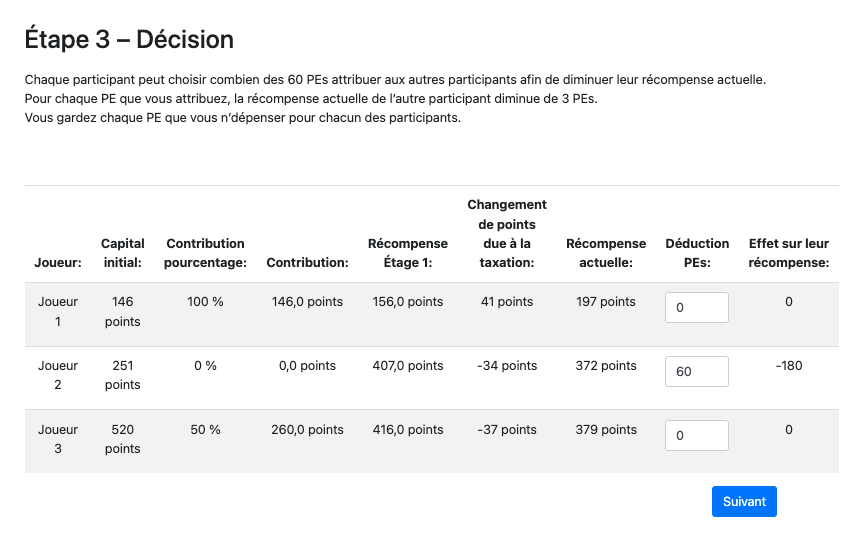


**Figure S14.** Step 3, Punishment Decision

# S5. References

Perugini M, Gallucci M, & Costantini G (2018) A practical primer to power analysis for simple experimental designs. *International Review of Social Psychology* 31(1).

Cohen J (2013) *Statistical power analysis for the behavioral sciences* (Routledge).
